# Baseline Assessment of Handwashing Behavior, Hand Hygiene Conditions, and Wellbeing in Primary Schools in Nigeria

**DOI:** 10.3389/ijph.2025.1608656

**Published:** 2025-09-25

**Authors:** Yaman Maani-Abuzahra, Mirko S. Winkler, Jan Hattendorf, Anaïs Galli, Andrea Tamas, Zainab Abdulkarim, Usman Modu Kolo, Muhammad Auwal Shuaibu, Maryna Peter, Nicole Probst-Hensch, Branwen N. Owen

**Affiliations:** ^1^ Department of Epidemiology and Public Health, Swiss Tropical and Public Health Institute, Allschwil, Switzerland; ^2^ University of Basel, Basel, Basel-Stadt, Switzerland; ^3^ Faculty of Medicine and Health Sciences, An-Najah National University, Nablus, Palestine; ^4^ Ranas Ltd., Zürich, Switzerland; ^5^ University of Applied Sciences and Arts, Northwestern Switzerland, Muttenz, Switzerland; ^6^ Terre des Hommes, Maiduguri, Nigeria

**Keywords:** hand hygiene, handwashing, WASH, wellbeing, schoolchildren

## Abstract

**Objectives:**

In humanitarian settings, poor school hygiene conditions can severely impact children’s health and wellbeing. As part of a cluster randomized trial evaluating a multicomponent hand hygiene intervention, this baseline study assessed hand hygiene behaviors, school infrastructure, and wellbeing among schoolchildren in Nigeria.

**Methods:**

Between May and June 2023, cross-sectional data were collected from 26 schools using handwashing observations, questionnaires, infrastructure assessments, and hand rinse sampling. A total of 964 children were observed, 645 interviewed, and 311 provided samples.

**Results:**

Observed handwashing rates were extremely low: 4%–12% before eating and 2%–3% after toilet use. About half of schools lacked designated handwashing stations. General water points, though more available, were often inadequate. Soap was entirely absent. Over half of children’s hands rinse samples contained more than 100 colony-forming units (CFU) of *Escherichia coli (E: coli)* per 100 mL. Misconceptions about hygiene were widespread and gaps existed between reported and observed behavior.

**Conclusion:**

These findings underscore the need for integrated school-based WASH interventions in humanitarian contexts.

## Introduction

Proper handwashing with soap is a well-documented intervention for reducing the spread of diarrheal diseases [1–4] and respiratory infections [5, 6]. Hand hygiene is also crucial for controlling epidemics such as cholera, Ebola, hepatitis E, severe acute respiratory syndrome (SARS), and COVID-19 [7–12]. Beyond disease prevention, handwashing helps reduce antibiotic use and supports efforts against antimicrobial resistance [13, 14]. This holds particularly true in humanitarian contexts, where poor access to water and hygiene has been shown to negatively affect not only physical health, but also psychological status and overall life satisfaction [15, 16].

Despite progress in increasing access to safe water and sanitation, billions of people—primarily in low- and middle-income countries (LMICs)—still lack access to essential water, sanitation and hygiene (WASH) services [17]. This also applies to Nigeria, where 151 million people are lacking basic hygiene services, defined as access to handwashing facilities with soap and water [18]. Nearly one-third of Nigerian children also lack sufficient water to meet their daily needs, further compounding the challenges to maintaining adequate hygiene [19]. Moreover, two-thirds of schools in Nigeria lack hygiene services, while nearly half lack sanitation facilities, defined as facilities that safely manage human excreta (urine and feces) to avoid human contact [20]. The situation is particularly severe in Borno State, where millions remain displaced due to conflict and climate-related events, further straining already weak school infrastructure and WASH systems [21, 22].

The Hands4Health (H4H) project responds to these needs by implementing a multicomponent hand hygiene intervention in schools in Borno State that lack essential WASH infrastructure and face overcrowding [23]. It promotes a systemic approach to hand hygiene and WASH in schools serving the most vulnerable populations while evaluating the effectiveness of the project’s intervention on schoolchildren’s hygiene practices and wellbeing through a cluster randomized controlled trial (cRCT).

This paper presents the baseline findings from the cRCT, offering a comprehensive overview of current school-based hand hygiene conditions, and children’s wellbeing and behaviors, assessed through handwashing observations, questionnaires, and hand rinse sampling. To our knowledge, this is the first study to directly observe schoolchildren’s handwashing behavior in Nigeria and to situate these findings within the broader context of WASH infrastructure and wellbeing in humanitarian settings. This adds valuable evidence to a largely understudied area and addresses an important child health issue in a fragile context.

## Methods

### Study Setting and Participants

**FIGURE 1:**
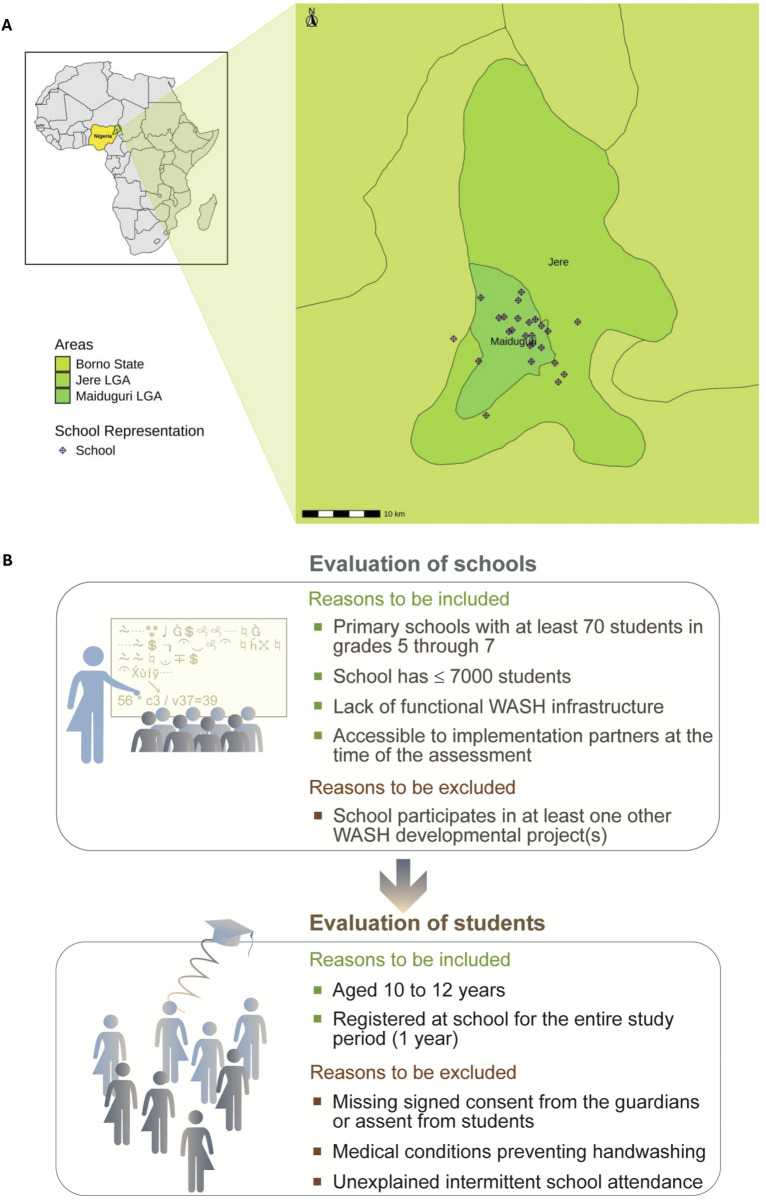
**(A)** Map of the study area showing the geographical distribution of primary schools included in the study; **(B)** Eligibility criteria for school and schoolchildren selection, including reasons for inclusion and exclusion (Baseline assessment of handwashing behavior, hand hygiene conditions, and wellbeing in primary schools, Jere and Maiduguri Metropolitan Council, Nigeria, May–June 2023).

The study was conducted in peri-urban and rural primary schools in Jere and Maiduguri Metropolitan Council (MMC) Local Government Areas (LGAs) in Maiduguri, Borno State (Figure 1A). Maiduguri was selected due to the presence of an NGO partner with prior WASH implementation experience. The city, Borno State’s capital, has experienced rapid population growth, largely driven by internally displaced persons (IDPs) fleeing the Boko Haram insurgency [24, 25]. Despite recent government efforts to close IDP camps, Maiduguri’s infrastructure remains under pressure, particularly in term of water access [26, 27]. Schools in the area continue to face overcrowding, understaffing, and insufficient WASH infrastructure [18].

### Study Design

This baseline assessment was conducted as part of a parallel two-arm cRCT conducted in primary schools across two LGAs in Maiduguri, Borno State (details provided in [23]). Schools were randomly allocated to intervention or control arms in a 1:1 ratio. Baseline data were collected between May and June 2023, using structured observations, questionnaires, and microbiological hand rinse sampling.

### Sample Size Calculation and Sampling Procedure

For the cRCT, we utilized the software R (version 4.1.3; R Foundation for Statistical Computing) to determine the required sample size for the clusters of schools and schoolchildren. The sample size calculation was based on achieving 80% statistical power and a 95% confidence level, with an assumed intraclass correlation coefficient of 0.2. It was estimated that the prevalence of handwashing before eating would be 20% in control schools, increasing to 45% in intervention schools 1 year post-baseline. From these assumptions, we estimated that 13 schools per trial arm, each including 50 schoolchildren, would be adequate to meet the study’s objectives. Although the sample size calculation was performed to assess power for the intervention effect [23], the number of children and clusters appears to be sufficient for the descriptive baseline analyses presented here.

### Recruitment and Eligibility

The recruitment of schools for the cRCT followed a three-step process. First, the Borno State Universal Basic Education Board in Nigeria identified schools in urgent need of WASH infrastructure improvements. Second, project implementation partners assessed these schools using the Facility Evaluation Tool for WASH in Schools (FACET WINS), a standardized monitoring tool for WASH services in schools [28]. Third, schools were evaluated against predefined inclusion criteria, as outlined in Figure 1B. Finally, 26 schools that met the criteria and demonstrated the greatest need for WASH improvements were selected for the study.

For the recruitment of schoolchildren, eligibility was determined based on criteria outlined in Figure 1B. Two classes from each school, with at least 70 eligible children, were randomly selected. From these classes, 50 eligible children per school were chosen using simple random sampling in Excel. Subsets for different data collection tools were then randomly selected from this group of 50 children. All 50 selected schoolchildren per school were required for structured handwashing observation, as it measured the primary outcome. However, for the other data collection tools, 25 schoolchildren per school were required for the questionnaire, and 12 schoolchildren per school for hand rinse sample collection, reflecting considerations related to data sufficiency, feasibility, and resource constraints.

### Randomization

We employed covariate-constrained randomization to balance key baseline characteristics between the intervention and control arms [23, 29].

### Data Collection Tools and Procedures

Baseline data collection utilized three tools (for details on the tools see [23]):

1) Covert structured observations of schoolchildren’s handwashing behavior, and handwashing facilities.
2) Questionnaire administered through interviews with schoolchildren.
3) Hand rinse sampling using a modified glove juice method, as described previously [30].

To minimize potential response bias, two different data collection teams administered these tools on separate days. On the first day, a team conducted covert observations for 3 h in each school without revealing their identity as data collectors. On the following day, a second team administered the questionnaire and collected hand rinse samples immediately afterwards.

### Measurement of Outcome Variables

#### Primary Outcome

The primary outcome was observed handwashing before eating, assessed in a structured setting to ensure comparability across children. To facilitate this, children participated in a painting activity followed by receiving a snack. Observers recorded whether children washed their hands before eating the snack. The outcome was calculated as the proportion of children who washed their hands out of the sample observed [23].

#### Secondary Outcomes

Several secondary outcomes were assessed to provide a more comprehensive understanding of hand hygiene behavior, infrastructure, and related factors. These include the following:

(i) Observed handwashing after using the toilet;
(ii) Self-reported frequency of handwashing before eating at school;
(iii) Self-reported frequency of handwashing after using the toilet at school;
(iv) Observed handwashing during other opportunities (i.e., any observed event of handwashing not related to structured before-snack and post-toilet opportunities);
(v) Self-reported key handwashing situations at school;
(vi) Observed handwashing steps as defined by the WHO for proper handwashing [31];
(vii) Self-reported handwashing steps as defined by the WHO for proper handwashing [31];
(viii) *Escherichia coli (E. coli)* colony-forming units (CFU) counts in hand rinse samples, with detection range between 3.5 and 1,050 CFU per sample collected from both hands of the child [30].
(ix) Hygiene knowledge;
(x) RANAS (Risks, Attitudes, Norms, Abilities, and Self-regulation) behavioral factors [32];
(xi) Observed school hand hygiene infrastructure and moment-specific access;
(xii) Self-reported access to hand hygiene services at school; and
(xiii) Wellbeing across six domains and overall score of the KINDL^®^ (Kinder Lebensqualität Fragebogen) quality of life (QoL) scale [33].

Details on the calculation of secondary outcomes are provided in Supplementary Table S1.

### Data Management and Statistical Analyses

To ensure standardization and consistency of data collection across all participating schools, the first author, with two co-authors, conducted training sessions for all data collection teams, co-facilitated by field supervisors from the partner NGO. Following the training, the tools and procedures were piloted in two schools, and the results were jointly reviewed by the first author, another co-author and the field supervisors. Based on this review, feedback and recommendations were provided to the teams to improve performance and data quality. During fieldwork, each team was assigned to a specific data collection tool (observation, questionnaire, or hand rinse) and school visit schedules were structured to maintain consistency. Observation teams visited the schools before questionnaire teams to preserve the undercover nature of the observations and minimize the risk of influencing children’s behavior through prior interviews. Using separate teams also helped avoid any association between the observations and the questionnaires. NGO supervisors accompanied the teams during school visits, facilitated access and coordination, and monitored data collection activities to help ensure adherence to protocols and data quality. Daily checks were conducted in collaboration with the first author to review completeness and consistency of the data.

Observers collected data using paper-based forms and cross-checked their records to eliminate duplicate observations of the same children. They subsequently merged observations made at different critical times or water points for the same child and transferred the data to Open Data Kit (ODK) Central (version 2023.3.1) on smartphones and tablets for storage and initial analysis. We checked the data collected using both paper and digital forms daily for completeness.

Another data collection team used smartphones and tablets to collect questionnaire responses and hand rinse samples directly through ODK Central. We conducted all analyses in R (version 4.3.2 [34]). Categorical and continuous variables were described as frequencies and percentages or means and standard deviations, respectively. The results are presented both as overall findings and stratified by intervention and control arms of the study.

### Ethics

Ethical approval for the study was granted by the National Health Research Ethics Committee in Nigeria (21/2023) and the Ethics Committee for Northwestern and Central Switzerland (Ethikkommission Nordwest-und Zentralschweiz; AO_2023-00047). The trial protocol is registered on ClinicalTrials.gov (NCT05964478). Approvals were also obtained from the Borno State Ministry of Education, Ministry of Health and the principals of the participating schools.

To address the covert nature of the observation, children could not be informed beforehand but were debriefed at the end of the study. Guardians provided written informed consent, which included information about the observational component, and participating schoolchildren provided oral assent. All participants were assigned unique identifiers, and data were fully anonymized to ensure confidentiality.

## Results

### Enrolment

Of the targeted sample of 50 randomly selected schoolchildren in each of the 26 schools, 964 children were present on the day of data collection and had their handwashing behavior observed. Randomly selected subsets of 645 children participated in the questionnaire and 311 provided hand rinse samples on the scheduled data collection days (Supplementary Figure S1).

### Participant Characteristics

Presented in Table 1 are the socio-demographic characteristics of 964 schoolchildren included in the study, stratified by trial arm. All participating children were enrolled in Grade 5. The majority (79%) were from MMC, with a similar distribution between control and intervention groups (Table 1). Girls comprised 58% of the sample, with comparable gender distribution across trial arms. The mean age of children was 11.3 years (SD = 0.9) with no substantial differences between trial arms.

**TABLE 1:** Socio-demographic characteristics of schoolchildren (N = 964) (Baseline assessment of handwashing behavior, hand hygiene conditions, and wellbeing in primary schools, Jere and Maiduguri Metropolitan Council, Nigeria, May–June 2023).

Characteristic	N (%)		
	Overall N = 964	Control N = 483	Intervention N = 481
Local Government Area			
Jere	199 (21%)	99 (20%)	100 (21%)
MMC	765 (79%)	384 (80%)	381 (79%)
Gender			
Female	562 (58%)	273 (57%)	289 (60%)
Male	402 (42%)	210 (43%)	192 (40%)
Age (years)			
Mean (SD)	11.3 (0.9)	11.2 (0.8)	11.3 (0.9)

### Handwashing Behavior of Schoolchildren

#### Observed Handwashing Before Eating a Snack During a Structured Opportunity (Primary Outcome)

**FIGURE 2:**
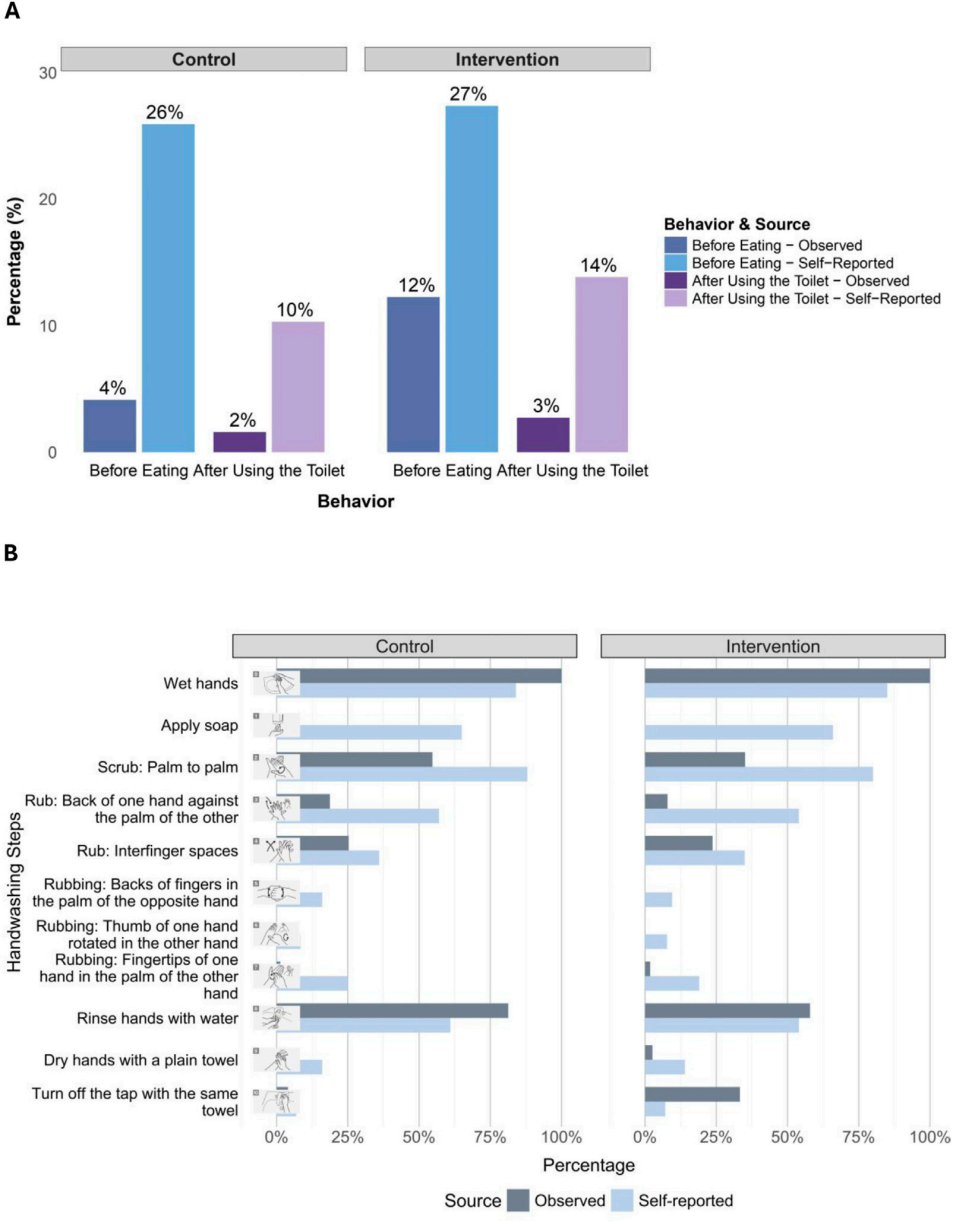
**(A)** Comparison of observed *versus* self-reported handwashing behavior before eating and after using the toilet among children in intervention and control schools; **(B)** Comparison of observed vs. self-reported usual handwashing steps across different handwashing moments among children in intervention and control schools (Baseline assessment of handwashing behavior, hand hygiene conditions, and wellbeing in primary schools, Jere and Maiduguri Metropolitan Council, Nigeria, May–June 2023).

Among the 964 schoolchildren we observed during the structured opportunity—handwashing before eating a provided snack— 8% washed their hands using water only (12% intervention, 4% control) (Figure 2A; Supplementary Table S2). Handwashing behavior seemed to be modestly clustered across schools (intraclass correlation coefficient = 0.14). Patterns were similar across sexes, with 9% of girls and 8% of boys washing their hands before eating (Supplementary Table S3).

#### Observed Handwashing After Using the Toilet

Out of 434 observed opportunities for toilet use among 404 children during the observation period, 2% involved handwashing using water only (3% in intervention schools and 2% in control schools) (Figure 2A; Supplementary Table S2). Very few children washed their hands after toilet use in either sex, with slightly higher rates observed among girls (3%) than boys (1%) (Supplementary Table S3).

#### Self-Reported Handwashing Frequency Before Eating and After Using the Toilet

Out of the 645 schoolchildren who reported about their handwashing behavior while at school, 26% of the control vs. 27% in the intervention groups said that they usually wash their hands before eating more than half of the times, while 10% of the control and 14% of the intervention said that they have the same frequency after using the toilet (Figure 2A). Sex-stratified results showed higher self-reported handwashing among girls than boys for both behaviors (i.e. 31% vs. 20% before eating; 14% vs. 9% after toilet use) (Supplementary Table S3).

Comparisons between self-reported and observed handwashing behaviors revealed gaps between the two methods. For handwashing before eating, 11% of children who reported washing their hands more than half of the time were actually observed doing so, while 7% of those who reported less frequent handwashing were nonetheless observed washing their hands. Similarly, for handwashing after toilet use, none of the 28 children who reported frequent handwashing were observed doing so, and 3 of 217 children who reported lower-frequency handwashing were observed washing their hands, as illustrated in the corresponding cross-tabulations and visualisations (Supplementary Table S4).

#### Additional Observed Handwashing During Other Opportunities

Beyond the two systematically assessed handwashing moments—before eating a provided snack and after using the toilet—we observed a total of 148 additional handwashing events during the observation period (52% in control, 48% in intervention) (Supplementary Table S5). Nearly half of these events occurred after eating (49% in control, 41% in intervention), while other opportunities were less frequent (Supplementary Table S5).

#### Self-Reported Usual Key Situations for Handwashing

Children also reported their usual key situations for handwashing at school (Supplementary Table S6). Before eating was the most frequently mentioned moment, reported by 84% of children in the control group and 78% in the intervention group (Supplementary Table S6).

#### Observed and Self-Reported Handwashing Steps

While children reported frequent handwashing at key moments, the quality of handwashing varied, with notable discrepancies between self-reported and observed handwashing steps (Figure 2B; Supplementary Table S7). We were able to observe the handwashing technique for only 189 children (40% control, 60% intervention) due to crowding, while 645 children (50% control, 50% intervention) provided self-reported data. These self-reports referred to their usual handwashing steps while at school. Soap use was entirely absent in observed handwashing, yet 66% of children reported applying it. Basic steps like palm-to-palm rubbing were more frequently reported (88% control, 80% intervention) than observed (55% control, 35% intervention), while more complex techniques, such as rubbing the backs of fingers or thumbs, were not observed (0%) but still self-reported (8%–16%) (Figure 2B; Supplementary Table S7).

### *E. coli* Contamination

**FIGURE 3:**
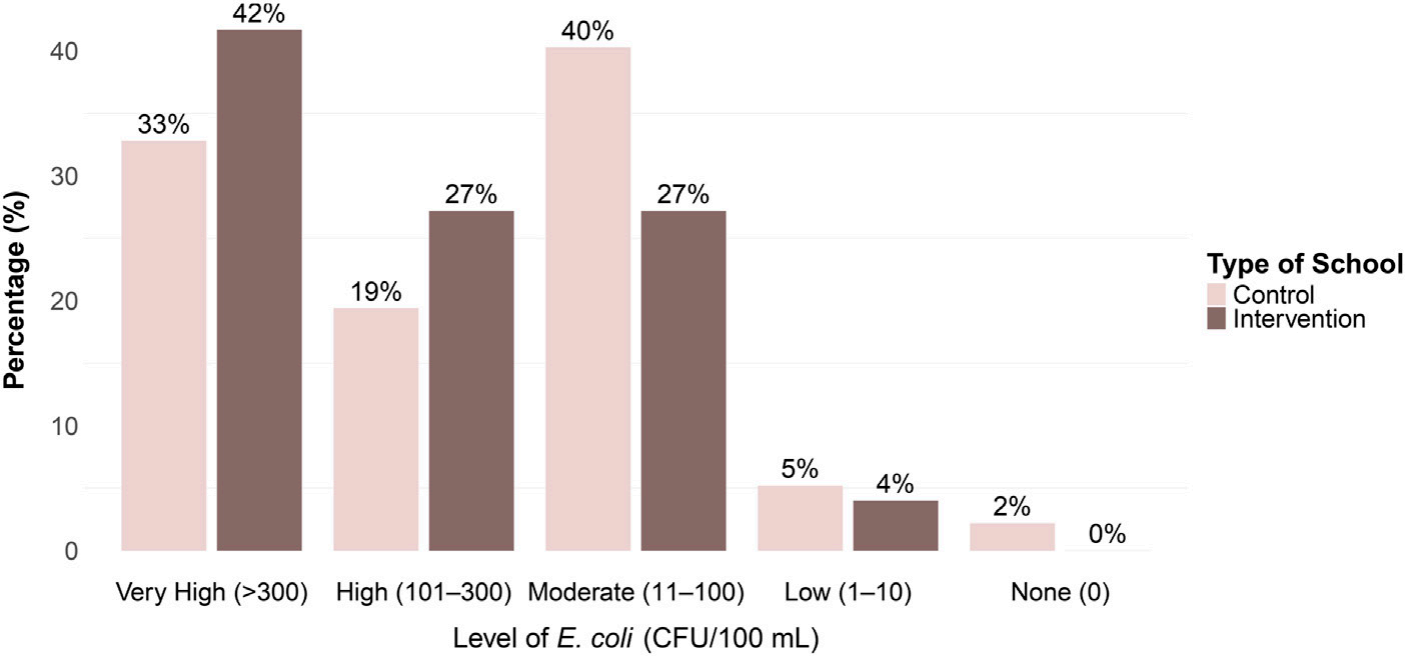
*Escherichia coli* (colony forming units/100 mL) levels in hand rinse samples from schoolchildren in intervention and control schools (Baseline assessment of handwashing behavior, hand hygiene conditions, and wellbeing in primary schools, Jere and Maiduguri Metropolitan Council, Nigeria, May–June 2023). Based on a total sample volume of 350 mL and a 100 mL filtration, the detection range was 3.5–1,050 colony forming units per sample (1–300 colony forming units per 100 mL). Each sample represents rinse water from both hands of a single child.

Most children had high levels of *E. coli* contamination on their hands, with 33% (control) and 40% (intervention) showing very high levels (>300 CFU/100 mL), and an additional 19% and 27%, respectively, in the high range (101–300 CFU/100 mL) (Figure 3; Supplementary Table S8). Only a small fraction (4%–5%) had low contamination levels (1–10 CFU/100 mL), and almost none had zero *E. coli* (Figure 3; Supplementary Table S8). Stratified analysis by sex revealed a similar distribution of *E. coli* levels, with boys showing a slightly higher proportion of very high contamination (>300 CFU/100 mL) compared to girls (41% vs. 36%) (Supplementary Table S3).

### Hygiene Knowledge and Behavioral Factors

The majority of children understood that touching dirty things transmits diseases (92% in control and 94% in intervention) and most of them recognized that germs can stick to hands (76% in control and 68% in intervention). However, 63% of children incorrectly believed that washing hands with water only is enough to remove germs, with similar distributions across groups (Supplementary Table S9).

While many children reported high perceived importance of hygiene, levels of risk perception, action control, and confidence in performing proper handwashing were mixed (Supplementary Table S10). Feelings of guilt for not washing hands were common, reported by 46% (control) and 48% (intervention) before eating, and 33% (control) and 38% (intervention) after using the toilet. However, self-monitoring of handwashing was lower than reported guilt for both moments, with 40% (control) and 45% (intervention) actively monitoring their handwashing before eating, and only 25% (control) and 34% (intervention) after using the toilet.

Barriers to handwashing were also reported, with 32% (control) and 31% (intervention) citing a lack of soap as a reason for not washing their hands. Forgetting was a common challenge, affecting 40% (control) and 35% (intervention) before eating, and 56% (control) and 50% (intervention) after using the toilet.

### School Hand Hygiene Conditions

#### Observed School Hand Hygiene Infrastructure Characteristics and Moment-Specific Access

Table 2 summarizes the observed availability and functionality of hand hygiene infrastructure across study schools. Handwashing stations (HWS)—dedicated water points for handwashing—were absent in 54% of intervention and 38% of control schools. Among schools with HWS, 50% (intervention) and 63% (control) had none meeting adequacy criteria (functional or semi-functional, with water available and located near toilets), while the rest had a very low ratio per 1,000 children. Soap was unavailable at all HWS.

**TABLE 2:** Hand hygiene infrastructure characteristics across schools (N = 26) (Baseline assessment of handwashing behavior, hand hygiene conditions, and wellbeing in primary schools, Jere and Maiduguri Metropolitan Council, Nigeria, May–June 2023).

Hand hygiene infrastructure characteristics	N (%)		
	Overall	Control	Intervention
Schools: HWS^a^ availability across schools	26	13	13
None (0 HWS per child)	12 (46%)	5 (38%)	7 (54%)
Very Low (<1 HWS per 1,000 children)	10 (38%)	6 (46%)	4 (31%)
Low (1 HWS per 500–1,000 children)	3 (12%)	2 (15%)	1 (8%)
Moderate (>1 HWS per 500 children)	1 (4%)	0 (0%)	1 (8%)
Schools with ≥1 HWS: *availability of HWS that are functional, having water and located near toilets, across schools* ^b^	14	8	6
None (0 HWS per child)	8 (57%)	5 (63%)	3 (50%)
Very Low (<1 HWS per 1,000 children)	6 (43%)	3 (38%)	3 (50%)
Schools with ≥1 HWS: *soap availability at available HWS across schools*	14	8	6
None (0 HWS with available soap per child)	14 (100%)	8 (100%)	6 (100%)
HWS: *type of available HWS*	38	18	20
Pipe and Tap	30 (79%)	15 (83%)	15 (75%)
Container-Based Sources	8 (21%)	3 (17%)	5 (25%)
Schools: GWP^a^ availability across schools	26	13	13
Very Low (≤1 GWP per 1,000 children)	8 (31%)	3 (23%)	5 (38%)
Low (1 GWP per 300–1,000 children)	14 (54%)	7 (54%)	7 (54%)
Moderate (1 GWP per 160–300 children)	3 (12%)	2 (15%)	1 (8%)
High (>1 GWP per 160 children)	1 (4%)	1 (8%)	0 (0%)
Schools with ≥1 GWP: *availability of GWP that are functiona, and having water across schools* ^c^	26	13	13
None (0 GWP per child)	3 (12%)	1 (8%)	2 (15%)
Very Low (<1 GWP per 1,000 children)	18 (69%)	10 (77%)	8 (62%)
Low (1 GWP per 300–1,000 children)	4 (15%)	2 (15%)	2 (15%)
Moderate (1 GWP per 160–300 children)	1 (4%)	0 (0%)	1 (8%)
Schools with ≥1 GWP: *soap availability at available GWP across schools*	26	13	13
None (0 GWP with available soap per child)	26 (100%)	13 (100%)	13 (100%)
GWP: Type of available GWP across schools	111	61	50
Pipe and Tap	76 (69%)	44 (72%)	32 (64%)
Borehole Pump	25 (22%)	12 (20%)	13 (26%)
Container-Based Sources	10 (9%)	5 (8%)	5 (10%)

^a^ Abbreviations: HWS, hand washing stations; GWP, general water points.

^b^ Functionality refers to HWS, that were either fully functional or functional but required some repairs. Water availability includes stations where water was available either continuously or intermittently during observation period. Location refers to HWS, situated inside sanitary units or positioned nearby.

^c^ Functionality refers to GWP, that were either fully functional or functional but required some repairs. Water availability includes GWP, where water was available either continuously or intermittently during the observation period.

General water points (GWP), which serve multiple purposes including handwashing, were more frequently available than HWS but were often inadequate. Most schools had low or very low availability of GWP, based on the number of points relative to the total number of students enrolled in the school. Moreover, 62% (intervention) and 77% (control) had a very low ratio of adequate GWP per 1,000 children, where adequacy was defined as being functional or semi-functional, with water available at the time of assessment. Soap was absent at all GWP. Pipe-and-tap systems were the most common source type.

As water availability varied during the day, moment-specific access was also recorded during observations. At the structured handwashing opportunity before eating a provided snack, 27% (control) and 33% (intervention) had access to HWS with water, and 92% and 86%, respectively, had access to GWP. During toilet use, 34% (both groups) had access to HWS with water, while access to GWP was 95% (control) and 93% (intervention) (Supplementary Table S2).

#### Self-Reported Access to Hand Hygiene Services at School

Almost half of the schoolchildren reported that the frequency of having sufficient water for handwashing at school was more than half of the times (44% in control, 55% in intervention). While 61% of the control group and 56% of the intervention group perceived the available handwashing water as generally clean, perceptions of specific water qualities varied (Supplementary Table S11). Overall satisfaction with school HWS was low, with only 42% of the control group and 34% of the intervention group rating their satisfaction above medium (Supplementary Table S11).

### Wellbeing of Schoolchildren

The mean total QoL scores across intervention and control groups were nearly identical (Table 3). Across QoL dimensions, emotional wellbeing was the highest-rated domain (71 in control, 70 in intervention), while self-esteem had the lowest scores (55 in control, 51 in intervention), followed by school functioning (58 in control, 57 in intervention) (Table 3). Other domains, including physical wellbeing, family connection, and social wellbeing (friends), showed similar patterns between groups, with minor variations. Stratified analysis by sex showed no substantial differences in QoL scores (Supplementary Table S12). Descriptive comparisons of wellbeing scores by observed and self-reported handwashing behaviors showed no consistent patterns or substantial differences across domains (Supplementary Table S13).

**TABLE 3:** Self-reported quality of life (QoL) of schoolchildren (N = 645) (Baseline assessment of handwashing behavior, hand hygiene conditions, and wellbeing in primary schools, Jere and Maiduguri Metropolitan Council, Nigeria, May–June 2023).

Quality of life	Mean (SD)		
	Overall N = 645	Control N = 320	Intervention N = 325
Total quality of life	65.2 (8.9)	65.4 (8.9)	65.0 (8.9)
Physical wellbeing	70.0 (17.0)	69.6 (17.3)	70.4 (17.1)
Emotional wellbeing	70.7 (17.1)	71.1 (17.1)	70.3 (17.1)
Self-esteem	52.9 (24.0)	54.5 (24.0)	51.4 (23.9)
Family connection	69.8 (17.4)	69.4 (18.1)	70.2 (16.8)
Friends (social wellbeing)	70.2 (17.9)	69.6 (17.8)	70.9 (18.0)
Functioning at school	57.4 (16.5)	57.8 (15.3)	57.0 (17.5)

^a^ Obtained from KINDL^®^ quality of life (QoL) scale. Scores were transformed to a 0–100 scale, with higher scores indicating better QoL.

## Discussion

To the best of our knowledge, this is the first study to assess handwashing behavior of primary schoolchildren in rural and peri-urban areas of Nigeria, particularly in a humanitarian setting, using a combination of three data collection methods. In addition to evaluating handwashing behavior, the study examined school hand hygiene infrastructure, children access and satisfaction with available hand hygiene conditions, psychosocial and behavioral determinants, and the overall wellbeing of children—factors that may be associated with hand hygiene practices. The findings highlight critical gaps in hand hygiene infrastructure, low observed and self-reported handwashing rates, a discrepancy between reported and observed behaviors, and poor school-related wellbeing—issues that reflect broader challenges in WASH conditions in Nigerian schools in Borno State.

Observations revealed that while many schools had some form of water access, dedicated HWS were often absent or, when available, were inadequate—non-functional, lacking water, or not located near toilets. Instead, children relied on GWP, which were more common but similarly constrained in functionality and accessibility. According to school staff, non-functionality was frequently attributed to broken or missing plastic taps, stemming from a combination of poor design quality, inadequate maintenance, heavy usage, and occasional vandalism. These infrastructural limitations, compounded by the complete absence of soap, created considerable barriers to proper hand hygiene. This was reflected in children’s self-reports, with nearly one-third identifying lack of soap as a key barrier. These challenges mirror findings from other LMIC contexts, where inadequate WASH infrastructure is often linked to poor infrastructure planning and limited resources [35–46]. Addressing these limitations requires investment in durable and child-friendly designs, reliable water supply systems, sustainable maintenance strategies and reliable supply chains for essential consumables such as soap, cleaning utensils, and detergents, as well as funding mechanisms to cover recurrent costs [47].

Severe infrastructure limitations, reflected in the very low satisfaction with hygiene facilities, likely contributed to the extremely low handwashing rates observed, particularly after toilet use. Although GWP were available to most children at observed moments, these were often inadequate for proper handwashing, and dedicated stations were rarely available. Thus, while access was not entirely absent, suboptimal conditions likely limited children’s ability to consistently wash their hands. Fewer than one in ten children washed their hands before eating, and almost none did so after using the toilet. Self-reported rates, while slightly higher, remained low, with only a quarter reporting handwashing before meals and just over one in ten after toilet use. Similar trends were observed in Guinea [35], yet our rates were even lower than those reported in other fragile school settings, where observed handwashing before eating ranged from 15% to 34% and after toilet use from 15% to 39% [48–50]. Self-reported handwashing was also higher in these studies, reaching up to 85% before eating and 83% after toilet use [37, 41, 51–56]. The exceptionally low rates of handwashing in this study underscore the need for multi-component interventions that combine infrastructure improvements with behavior change strategies and stronger institutional support within the school system.

The higher handwashing rates before eating compared to after toilet use align with findings from previous studies, which also reported greater compliance with handwashing before meals than after toilet use among schoolchildren [51, 53–56]. This pattern may reflect a greater emphasis on handwashing before meals or the poor sanitation conditions and limited proximity of HWS to school toilets. Behavioral insights further suggest that social norms and self-regulatory factors may play a role: children reported stronger feelings of guilt and higher self-monitoring for missed handwashing before meals, while forgetting was more commonly cited as a barrier after toilet use. This behavioral gap was reflected in the *E. coli* contamination levels, with more than half of children having high bacterial loads on their hands, Only a few studies have reported such high concentrations in school settings [55, 57, 58]. These findings highlight the urgent need to prioritize safe, child-friendly sanitation, ensure HWS are physically accessible, particularly near toilets, and address behavioral factors that foster habit formation, such as providing timely reminders and leveraging social motivators to support handwashing at all critical moments.

Agreement between self-reported and observed handwashing behaviors was low for both moments—before eating and after toilet use—with particularly pronounced discrepancies after toilet use. While 11% of children who reported frequent handwashing before eating were actually observed doing so, none of those who reported frequent handwashing after toilet use were observed washing their hands. These findings are consistent with previous studies in school settings, where self-reports have been shown to overestimate hand hygiene [59, 60]. This likely reflects over-reporting due to social desirability or recall bias and highlights the importance of using multiple data collection methods to assess hygiene behavior in school settings more reliably.

Our sex-stratified analysis of hygiene behaviors and *E. coli* contamination revealed no substantial differences between boys and girls, suggesting that poor hand hygiene and fecal contamination were widespread, regardless of sex. This aligns with other studies in low-resource school settings where gender differences in hygiene behavior are often less pronounced in pre-puberty age groups [61].

Furthermore, while many children demonstrated good knowledge of hand hygiene, there were misconceptions about effective handwashing. Nearly two-thirds incorrectly believed that washing with water alone is sufficient to remove germs, which may also indicate a lack of appreciation for proper handwashing steps. This was reflected in observed behavior, where basic techniques like palm-to-palm rubbing were reported nearly twice as often as they were observed. A similar gap between knowledge and practice has been observed in other studies [40, 53, 62, 63], suggesting that this discrepancy may stem from both behavioral and infrastructure barriers. In our findings, although most children recognised the importance of hygiene, fewer demonstrated strong action control or self-regulation, with less than half actively monitoring their behavior at key moments. These findings highlight the importance of coupling hygiene education with context-specific behavior change strategies that target psychosocial factors and are backed by conducive environments to translate knowledge into consistent, effective behavior.

The challenging school environment, including poor WASH facilities, not only affects children’s handwashing practices but may also influence their overall wellbeing. Assessing children’s QoL revealed poor to moderate overall and domain-specific wellbeing scores, with self-esteem and school-related wellbeing showing the lowest scores. This may reflect the compounded effects of inadequate hygiene facilities, overcrowded and under-resourced classrooms [64, 65], and broader socioeconomic adversities. Factors such as political instability, insecurity, and poor housing conditions are widely known to affect child wellbeing in crisis-affected settings [66, 67]. Our sex-stratified analysis showed no substantial differences in QoL scores between boys and girls. This aligns with literature suggesting that gender gaps in wellbeing tend to emerge during adolescence, often linked to puberty-related changes [68, 69]. While few studies have explicitly measured QoL among schoolchildren in this region, the inclusion of wellbeing in this study offers a valuable reference point. In our descriptive baseline analysis, we found no consistent differences in wellbeing scores between children who were observed washing their hands and those who were not, nor between those who reported frequent handwashing and those who reported doing so less frequently. However, the relationship between hygiene conditions and behaviors and wellbeing remains underexplored in comparable fragile settings. Planned follow-up analyses from this cluster randomized trial will assess whether improvements in WASH infrastructure and hygiene behavior contribute to better QoL outcomes over time.

This study has several strengths. It was conducted in a challenging humanitarian setting, providing valuable evidence on handwashing behavior and WASH conditions in overcrowded, under-resourced schools. The use of multiple data collection tools, including direct observation, self-reports, infrastructure assessments, and microbiological analysis, enabled a comprehensive assessment. The intraclass correlation coefficient for the primary outcome (observed handwashing before eating) was 0.14, indicating lower-than-expected clustering and enhancing the precision of our estimates. However, some limitations should be noted. Although most children had access to GWP during observed handwashing moments, these facilities were often inadequate, and dedicated HWS were rarely available. These suboptimal conditions may have constrained children’s ability to wash their hands and should be considered when interpreting the low observed handwashing rates. Also, self-reported data, collected through face-to-face interviews, may be subject to social desirability bias, as individuals tend to overstate their hygiene practices in interviews [59, 60, 70]. This highlights the added value of using direct observation to obtain more reliable behavioral data. Additionally, only a limited number of children’s handwashing techniques could be fully observed due to overcrowding at water points, and efforts to maintain covert observation reduced opportunities for detailed technique assessment. Covert observation was employed to minimize reactivity and better capture children’s typical behavior. Data collectors were trained to remain discreet and avoid drawing attention, and no identifiable information was collected. However, despite these efforts, there remains a possibility that some children may have suspected the presence of foreign observers and altered their behavior accordingly. Despite these limitations, the study offers a strong baseline for evaluating future interventions.

### Conclusion

This baseline study examines hand hygiene behaviors, infrastructure, and contextual factors among primary schoolchildren in humanitarian settings in Borno State, Nigeria. Findings reveal extremely low handwashing rates at school, alongside inadequate infrastructure, intermittent water supply, lack of soap, and behavioral challenges. Addressing these issues requires comprehensive, school-based interventions that integrate infrastructure improvements with context-specific behavior change strategies and more substantial institutional commitment to support schools. This study lays a foundation for evaluating the H4H project’s intervention and guiding WASH-related school health policies.
