# Supplementary material for: Baseline Assessment of Handwashing Behavior, Hand Hygiene Conditions, and Wellbeing in Primary Schools in Nigeria
Source: Int J Public Health. 2025 Sep 25;70:1608656. doi: 10.3389/ijph.2025.1608656 (PMC12507709; doi:10.3389/ijph.2025.1608656)
Supplement: Supplementary file 1 [file DataSheet1.zip › Supplementary Table 1_revised.docx]

International Journal of Public Health

Baseline Assessment of Handwashing Behavior, Hand Hygiene Conditions, and Well-being in Primary Schools in Nigeria

## **Supplementary Table 1. Definition of the study’s secondary outcomes (Baseline assessment of handwashing behavior, hand hygiene conditions, and wellbeing in primary schools, Jere and Maiduguri Metropolitan Council, Nigeria, May–June 2023)**

| The outcome | Measurement method |
| --- | --- |
| Observed handwashing after using the toilet | Proportion calculated as the number of children who washed their hands after using the toilet divided by the number of children who used the toilet |
| Self-reported frequency of handwashing before eating | Proportion was defined as the number of children who self-reported washing their hands “more than half of the time” before eating divided by the total number of children interviewed |
| Self-reported frequency of handwashing after using the toilet | Proportion was defined as the number of children who self-reported washing their hands “more than half of the time” after using the toilet divided by the total number of children interviewed |
| Observed handwashing at other opportunities including before eating [excluding post-painting activity], after eating, after playing, or for no specific reason | Proportion calculated as the number of children who washed their hands during each opportunity divided by the total number of children observed for washing their hands for these opportunities |
| Self-reported key handwashing situations | Proportion was calculated as the number of children who mentioned specific key situations for handwashing (e.g., after entering the schoolyard, after playing, before eating, after using the toilet, after sweeping) divided by the total number of children interviewed |
| Observed handwashing steps as defined by the WHO for proper handwashing*^1^* | Proportion of each handwashing step was calculated as the number of children who practiced each step divided by the total number of children who washed their hands and their handwashing technique could be observed |
| Self-reported handwashing steps as defined by the WHO for proper handwashing*^1^* | Proportion of each handwashing step was calculated as the number of children who self-reported performing the step divided by the total number of children interviewed |
| *Escherichia coli* (*E. coli) c*ontamination | Proportion was calculated as the proportion of children within each *E. coli* concentration category, divided by the total number of children whose samples were analyzed and had zero or countable CFU counts |
| Hygiene knowledge: | Proportion of hygiene knowledge was defined as the number of children providing correct answers to hygiene-related questions divided by the total number of children interviewed |
| RANAS behavioral factors | Proportion was assessed based on self-reported responses using a 5-point Likert scale measuring the frequency or magnitude of these factors. Proportion was defined as the number of children who reported a frequency of “more than half the time” or a magnitude of “greater than medium,” divided by the total number of children interviewed |
| Observed school hand hygiene infrastructure and moment-specific access | Proportions were measured for observed infrastructure characteristics including HWS^2^ and GWP^2^ facilities   - Proportion of schools falling into each category of HWS and GWP availability (none, very low, low, moderate, high), calculated as the number of schools in each category divided by the total number of schools. - Proportion of schools with adequate HWS and GWP (defined as functional or semi-functional, with available water and [for HWS] located near toilets), categorized similarly and divided by the total number of schools - Proportion of HWS and GWP with soap availability, categorized as none, very low, low, moderate, or high, calculated by dividing the number of facilities in each category by the total number of HWS and GWP, respectively. - Proportion of each type of HWS and GWP (e.g., pipe-and-tap, container-based, borehole), calculated as the number of each type divided by the total number of respective facilities.   Proportion of observed access to hand hygiene services at specific moments (before eating a provided snack and post-toilet use)   - Proportion of children with access to HWS and GWP with available water at each observed moment, calculated as the number of children with access divided by the total number of children observed for that moment. |
| Self-reported access to hand hygiene services at school | Proportion was calculated as the number of children who self-reported a frequency of “more than half the time” or a magnitude of “more than medium” to a set of questions about access to handwashing services at school divided by the total number of children interviewed |
| Well-being | Mean of each of domain and overall score of the KINDL® quality of life (QoL) scale. The questionnaire consists of 24 items answered on a five-point Likert scale (1 = “always” to 5 = “never”), with higher scores indicating better QoL. Each item corresponds to one of six dimensions. Dimension scores were calculated as the sum of their respective items (range: 4–20) and transformed to a 0–100 scale. The overall QoL score was derived as the sum of all 24 items, also transformed to a 0-100 scale |

*^1^* [*https://www.who.int/docs/default-source/patient-safety/how-to-handwash-poster.pdf*](https://www.who.int/docs/default-source/patient-safety/how-to-handwash-poster.pdf)

^2^ HWS: Handwashing stations, GWP: General water points
