# Supplementary material for: Baseline Assessment of Handwashing Behavior, Hand Hygiene Conditions, and Wellbeing in Primary Schools in Nigeria
Source: Int J Public Health. 2025 Sep 25;70:1608656. doi: 10.3389/ijph.2025.1608656 (PMC12507709; doi:10.3389/ijph.2025.1608656)
Supplement: Supplementary file 1 [file DataSheet1.zip › Supplementary Table 2_revised.docx]

International Journal of Public Health

Baseline Assessment of Handwashing Behavior, Hand Hygiene Conditions, and Well-being in Primary Schools in Nigeria

## **Supplementary Table 2. Summary of the observed two critical moments for handwashing; before eating (post a provided snack) and after using the toilets** **for children in intervention and control schools) (Baseline assessment of handwashing behavior, hand hygiene conditions, and wellbeing in primary schools, Jere and Maiduguri Metropolitan Council, Nigeria, May–June 2023)**

|  | Before eating (post a provided snack) | | | After using the toilet | | |
| --- | --- | --- | --- | --- | --- | --- |
|  | **N (%)** | | | **N (%)** | | |
|  | Overall  N = 964 | Control  N = 483 | Intervention  N = 481 | Overall  N = 434 | Control  N = 251 | Intervention  N = 183 |
| Handwashing opportunities observed | 964 | 483 | 481 | 434 | 251 | 183 |
| Handwashing with water only | 79 (8%) | 20 (4%) | 59 (12%) | 9 (2%) | 4 (2%) | 5 (3%) |
| Water availability^1^ at HWS^2^ during the opportunity |  |  |  |  |  |  |
| Available all day | 176 (18%) | 99 (20%) | 77 (16%) | 92 (21%) | 58 (23%) | 34 (19%) |
| Partially available | 113 (12%) | 33 (7%) | 80 (17%) | 55 (13%) | 28 (11%) | 27 (15%) |
| Unavailable | 675 (70%) | 351 (73%) | 324 (67%) | 287 (66%) | 165 (66%) | 122 (67%) |
| Soap availability at HWS during the opportunity |  |  |  |  |  |  |
| Unavailable | 964 (100%) | 483 (100%) | 481 (100%) | 434 (100%) | 251 (100%) | 183 (100%) |
| Water availability^1^ at GWP^2^ during the opportunity |  |  |  |  |  |  |
| Available all day | 100 (10%) | 21 (4%) | 79 (16%) | 39 (9%) | 23 (9%) | 16 (9%) |
| Partially available | 762 (79%) | 426 (88%) | 336 (70%) | 371 (86%) | 216 (86%) | 155 (85%) |
| Unavailable | 102 (11%) | 36 (8%) | 66 (14%) | 24 (6%) | 12 (5%) | 12 (7%) |
| Soap availability at GWP during the activity |  |  |  |  |  |  |
| Unavailable | 964 (100%) | 483 (100%) | 481 (100%) | 434 (100%) | 251 (100%) | 183 (100%) |
| Among handwashing events | 79 | 20 | 59 | 9 | 4 | 5 |
| Soap availability at the place of the handwashing |  |  |  |  |  |  |
| Unavailable | 79 (100%) | 20 (100%) | 59 (100%) | 9 (100%) | 4 (100%) | 5 (100%) |
| Location of handwashing |  |  |  |  |  |  |
| GWP | 70 (89%) | 18 (90%) | 52 (88%) | 3 (33%) | 0 (0%) | 3 (60%) |
| HWS | 4 (5%) | 0 (0%) | 4 (7%) | 2 (22%) | 1 (25%) | 1 (20%) |
| Water bottle | 4 (5%) | 2 (10%) | 2 (3%) | 4 (44%) | 3 (75%) | 1 (20%) |
| Water sachet | 1 (1%) | 0 (0%) | 1 (2%) | 0 (0%) | 0 (0%) | 0 (0%) |
| Among handwashing events at HWS: | 4 | 0 | 4 | 2 | 1 | 1 |
| Type of HWS where handwashing took place |  |  |  |  |  |  |
| Bucket with tap | 4 (100%) | - | 4 (100%) | 0 (0%) | 0 (0%) | 0 (0%) |
| Pipe with taps | 0 (0%) | - | 0 (0%) | 1 (50%) | 0 (0%) | 1 (100%) |
| Large container with a scoop or a cup | 0 (0%) | - | 0 (0%) | 1 (50%) | 1 (100%) | 0 (0%) |
| Presence of a GWP with available water closer to the student than the HWS where the handwashing took place | 4 (100%) | - | 4 (100%) | 0 (0%) | 0 (0%) | 0 (0%) |
| Among handwashing events at GWP: | 70 | 18 | 52 | 3 | 0 | 3 |
| Type of GWP where handwashing took place |  |  |  |  |  |  |
| Pipe with taps | 42 (60%) | 6 (33%) | 36 (69%) | 3 (100%) | - | 3 (100%) |
| Large container with a scoop or a cup | 5 (7%) | 2 (11%) | 3 (6%) | 0 (0%) | - | 0 (0%) |
| Borehole pump | 23 (33%) | 10 (56%) | 13 (25%) | 0 (0%) | - | 0 (0%) |
| Presence of a HWS with available water closer to the students than the GWP where the handwashing took place | 6 (9%) | 1 (6%) | 5 (10%) | 0 (0%) | - | 0 (0%) |

^1^Water availability: Available all day; water was present at all HWS or GWP throughout the observation period, Partially available; water was intermittently available during observations, or some HWS/GWP had water while others did not, Unavailable; no water was available at any HWS or GWP at any time during the observation period.

^2^Abbreviations: HWS, Hand washing stations; GWP, General water points.
