# Supplementary material for: Baseline Assessment of Handwashing Behavior, Hand Hygiene Conditions, and Wellbeing in Primary Schools in Nigeria
Source: Int J Public Health. 2025 Sep 25;70:1608656. doi: 10.3389/ijph.2025.1608656 (PMC12507709; doi:10.3389/ijph.2025.1608656)
Supplement: Supplementary file 1 [file DataSheet1.zip › Supplementary Table 3_revised.docx]

International Journal of Public Health

Baseline Assessment of Handwashing Behavior, Hand Hygiene Conditions, and Well-being in Primary Schools in Nigeria

**Supplementary Table 3. Observed, self-reported behaviors and *Escherichia coli* (*E.coli*) levels stratified by sex of children in schools (Baseline assessment of handwashing behavior, hand hygiene conditions, and wellbeing in primary schools, Jere and Maiduguri Metropolitan Council, Nigeria, May–June 2023)**

|  | **N (%)** | | |
| --- | --- | --- | --- |
|  | Overall | Female | Male |
| **Observed handwashing behavior** |  |  |  |
| Observed handwashing before eating (post a provided snack) | N = 964  79 (8%) | N = 562  49 (9%) | N = 402  30 (8%) |
| Observed handwashing after using toilet | N = 434  9 (2%) | N = 275  8 (3%) | N = 159  1 (1%) |
| **Self-reported handwashing behavior** | N = 645 | N = 388 | N = 257 |
| Self-reported handwashing frequency before eating (more than half of the times) | 172 (27%) | 121 (31%) | 51 (20%) |
| Self-reported handwashing frequency after using toilet (more than half of the times) | 78 (12%) | 56 (14%) | 22 (9%) |
| ***Escherichia coli* (*E.coli*) level (CFU/100mL)** | N = 285 | N = 169 | N = 116 |
| None (0) | 3 (1%) | 1 (1%) | 2 (2%) |
| Low (1–10) | 13 (5%) | 7 (4%) | 6 (5%) |
| Moderate (11–100) | 95 (33%) | 59 (35%) | 36 (31%) |
| High (101–300) | 67 (24%) | 42 (25%) | 25 (22%) |
| Very high (> 300) | 107 (38%) | 60 (36%) | 47 (41%) |

*Abbreviations: CFU/100mL: colony-forming units/100 mL hand rinse samples*
