# Supplementary material for: Baseline Assessment of Handwashing Behavior, Hand Hygiene Conditions, and Wellbeing in Primary Schools in Nigeria
Source: Int J Public Health. 2025 Sep 25;70:1608656. doi: 10.3389/ijph.2025.1608656 (PMC12507709; doi:10.3389/ijph.2025.1608656)

International Journal of Public Health

Baseline Assessment of Handwashing Behavior, Hand Hygiene Conditions, and Well-being in Primary Schools in Nigeria

## **Supplementary Table 4. Comparison of observed and self-reported handwashing behavior before eating and after toilet use among children in schools (Baseline assessment of handwashing behavior, hand hygiene conditions, and wellbeing in primary schools, Jere and Maiduguri Metropolitan Council, Nigeria, May–June 2023)**

A. Comparison of observed and self-reported handwashing behavior before eating

|  | **Didn’t wash (obs)**  **N (%)** | **Washed (obs)**  **N (%)** | **Total**  **N (%)** |
| --- | --- | --- | --- |
| **Wash half or less than half of the times (self)** | 367 (93%) | 29 (7%) | 396 (100%) |
| **Wash more than half of the times (self)** | 132 (89%) | 17 (11%) | 149 (100%) |
| **Total** | 499 (92%) | 46 (8%) | 545 (100%) |


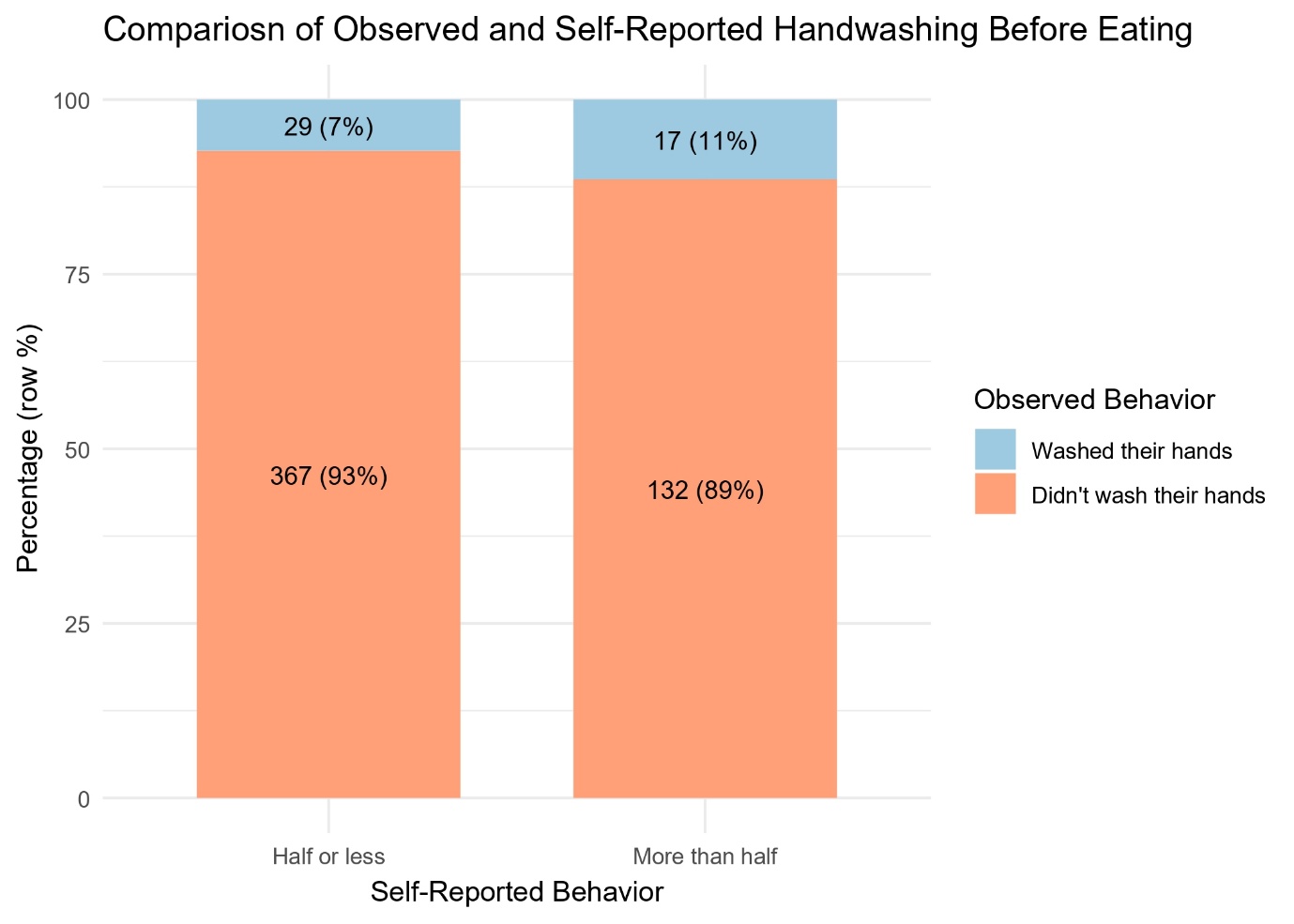


B. Comparison of observed and self-reported handwashing behavior after toilet use

|  | **Didn’t wash (obs)**  **N (%)** | **Washed (obs)**  **N (%)** | **Total**  **N (%)** |
| --- | --- | --- | --- |
| **Wash half or less than half of the times (self)** | 214 (99%) | 3 (1%) | 217 (100%) |
| **Wash more than half of the times (self)** | 28 (100%) | 0 (0%) | 28 (100%) |
| **Total** | 242 (99%) | 3 (1%) | 245 (100%) |


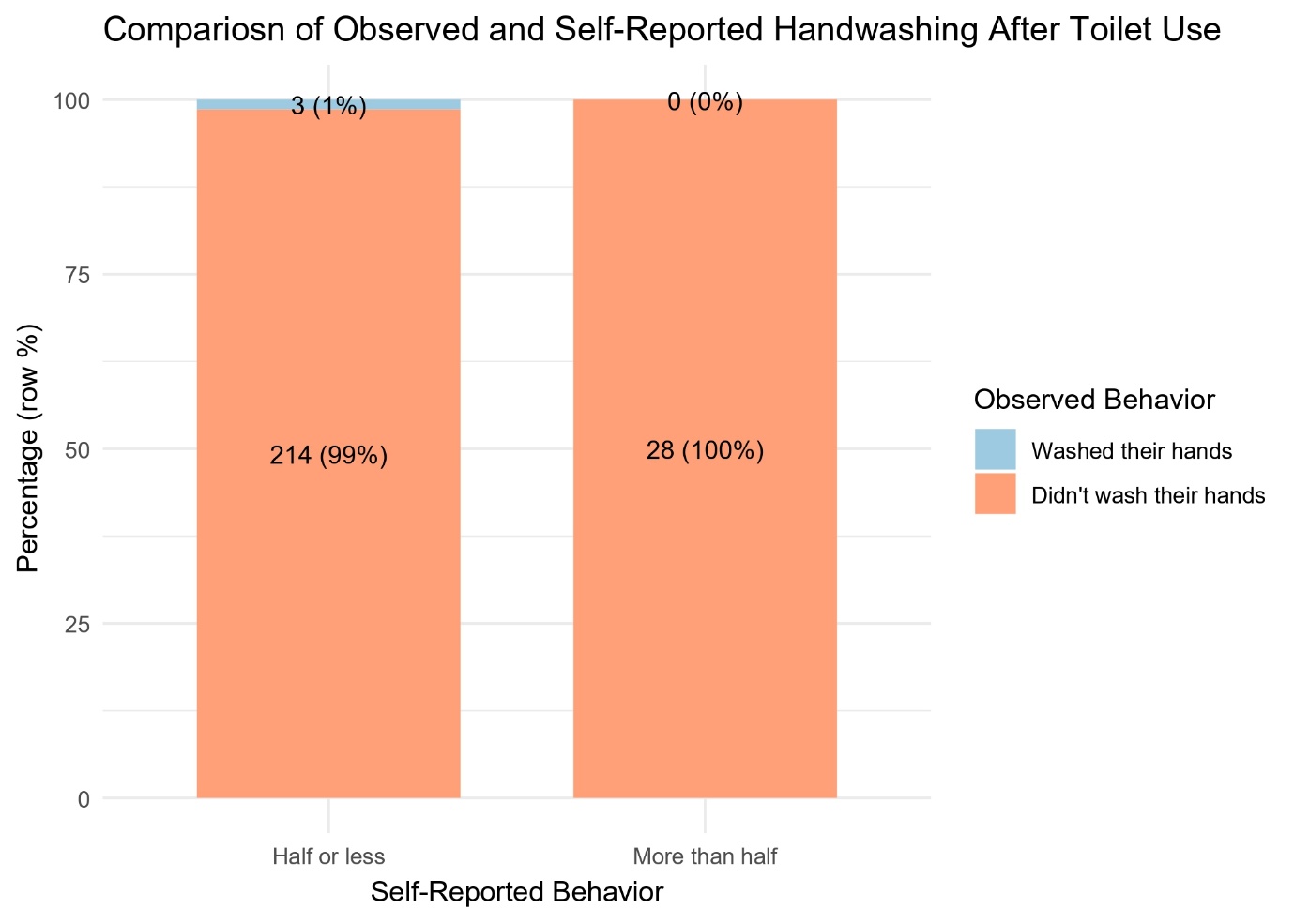

Supplement: Supplementary file 1 [file DataSheet1.zip › Supplementary Table 4_revised.docx]
