# Supplementary material for: Baseline Assessment of Handwashing Behavior, Hand Hygiene Conditions, and Wellbeing in Primary Schools in Nigeria
Source: Int J Public Health. 2025 Sep 25;70:1608656. doi: 10.3389/ijph.2025.1608656 (PMC12507709; doi:10.3389/ijph.2025.1608656)
Supplement: Supplementary file 1 [file DataSheet1.zip › Supplementary Table 5_revised.docx]

International Journal of Public Health

Baseline Assessment of Handwashing Behavior, Hand Hygiene Conditions, and Well-being in Primary Schools in Nigeria

## **Supplementary Table 5. Summary of additional observed handwashing events** **among children in intervention and control schools (Baseline assessment of handwashing behavior, hand hygiene conditions, and wellbeing in primary schools, Jere and Maiduguri Metropolitan Council, Nigeria, May–June 2023)**

|  | N (%) | | |
| --- | --- | --- | --- |
|  | Overall  N = 148 | Control  N = 77 | Intervention  N = 71 |
| Handwashing opportunity details | 148 | 77 | 71 |
| The type of the handwashing events |  |  |  |
| Before eating | 48 (32%) | 31 (40%) | 17 (24%) |
| After playing | 29 (20%) | 7 (10%) | 22 (31%) |
| After eating | 67 (45%) | 38 (49%) | 29 (41%) |
| No specific reason | 4 (3%) | 1 (1%) | 3 (4%) |
| Location of handwashing |  |  |  |
| GWP^1^ | 113 (76%) | 49 (64%) | 64 (90%) |
| HWS^1^ | 14 (10%) | 9 (12%) | 5 (7%) |
| Water bottle | 18 (12%) | 16 (21%) | 2 (3%) |
| Water sachet | 3 (2%) | 3 (4%) | 0 (0%) |
| Soap availability at the place of the handwashing location |  |  |  |
| Unavailable | 148 (100%) | 77 (100%) | 71 (100%) |
| Water availability2 at HWS during the opportunity |  |  |  |
| Available | 69 (47%) | 41 (53%) | 28 (39%) |
| Partially available | 22 (15%) | 0 (0%) | 22 (31%) |
| Unavailable | 57 (39%) | 36 (47%) | 21 (30%) |
| Soap availability at HWS during the opportunity |  |  |  |
| Unavailable | 148 (100%) | 77 (100%) | 71 (100%) |
| Water availability2 at GWP during the opportunity |  |  |  |
| Available | 47 (32%) | 16 (21%) | 31 (44%) |
| Partially available | 98 (66%) | 58 (75%) | 40 (56%) |
| Unavailable | 3 (2%) | 3 (4%) | 0 (0%) |
| Soap availability at GWP during the activity |  |  |  |
| Unavailable | 148 (100%) | 77 (100%) | 71 (100%) |
| Among handwashing events at HWS: | 14 | 9 | 5 |
| Type of HWS where handwashing took place |  |  |  |
| Bucket with tap | 4 (29%) | 0 (0%) | 4 (80%) |
| Pipe with taps | 1 (7%) | 0 (0%) | 1 (20%) |
| Large container with a scoop or a cup | 9 (64%) | 9 (100%) | 0 (0%) |
| Presence of a GWP with available water closer to the children than the HWS where the handwashing took place | 14 (100%) | 9 (100%) | 5 (100%) |
| Among handwashing events at GWP: | 113 | 49 | 64 |
| Type of GWP where handwashing took place |  |  |  |
| Pipe with taps | 72 (64%) | 22 (45%) | 50 (78%) |
| Large container with a scoop or a cup | 18 (16%) | 17 (35%) | 1 (2%) |
| Borehole pump | 23 (20%) | 10 (20%) | 13 (20%) |
| Presence of a HWS with available water closer to the children than the GWP where the handwashing took place | 10 (9%) | 8 (16%) | 2 (3%) |

^1^*Abbreviations: HWS, Hand washing stations; GWP, General water points*

^2^*Water availability: Available all day; water was present at all HWS or GWP throughout the observation period, Partially available; water was intermittently available during observations, or some HWS/GWP had water while others did not, Unavailable; no water was available at any HWS or GWP at any time during the observation period.*
