# Supplementary material for: Baseline Assessment of Handwashing Behavior, Hand Hygiene Conditions, and Wellbeing in Primary Schools in Nigeria
Source: Int J Public Health. 2025 Sep 25;70:1608656. doi: 10.3389/ijph.2025.1608656 (PMC12507709; doi:10.3389/ijph.2025.1608656)
Supplement: Supplementary file 1 [file DataSheet1.zip › Supplementary Table 6_revised.docx]

International Journal of Public Health

Baseline Assessment of Handwashing Behavior, Hand Hygiene Conditions, and Well-being in Primary Schools in Nigeria

## **Supplementary Table 6. Self-reported usual key situations for handwashing** **of children in intervention and control schools (Baseline assessment of handwashing behavior, hand hygiene conditions, and wellbeing in primary schools, Jere and Maiduguri Metropolitan Council, Nigeria, May–June 2023)**

|  | **N (%)** | | |
| --- | --- | --- | --- |
| **Usual key situations for handwashing** | Overall  N = 645 | Control  N = 320 | Intervention  N = 325 |
| After entering the schoolyard | 29 (5%) | 13 (4%) | 16 (5%) |
| After playing | 135 (21%) | 62 (19%) | 73 (23%) |
| Before eating | 522 (81%) | 269 (84%) | 253 (78%) |
| After using the toilet | 219 (34%) | 120 (38%) | 99 (30%) |
| After sweeping | 96 (15%) | 49 (15%) | 47 (14%) |
| **Additional key situations for handwashing mentioned by students** |  | | |
| After entering the schoolyard | 7 (1%) | 1 (0%) | 6 (2%) |
| After playing | 104 (16%) | 46 (14%) | 58 (18%) |
| Before eating | 104 (16%) | 46 (14%) | 58 (18%) |
| After using the toilet | 165 (26%) | 90 (28%) | 75 (23%) |
| After sweeping | 76 (12%) | 33 (10%) | 43 (13%) |
