# Supplementary material for: Baseline Assessment of Handwashing Behavior, Hand Hygiene Conditions, and Wellbeing in Primary Schools in Nigeria
Source: Int J Public Health. 2025 Sep 25;70:1608656. doi: 10.3389/ijph.2025.1608656 (PMC12507709; doi:10.3389/ijph.2025.1608656)
Supplement: Supplementary file 1 [file DataSheet1.zip › Supplementary Table 7_revised.docx]

International Journal of Public Health

Baseline Assessment of Handwashing Behavior, Hand Hygiene Conditions, and Well-being in Primary Schools in Nigeria

## **Supplementary Table 7. Summary of observed and self-reported handwashing steps of children in intervention and control schools (Baseline assessment of handwashing behavior, hand hygiene conditions, and wellbeing in primary schools, Jere and Maiduguri Metropolitan Council, Nigeria, May–June 2023)**

|  | **Observed steps** | | | **Self-reported steps** | | |
| --- | --- | --- | --- | --- | --- | --- |
|  | **N (%)** | | | **N (%)** | | |
| **Opportunity steps details** | Overall  N = 189 | Control  N = 75 | Intervention  N = 114 | Overall  N = 645 | Control  N = 320 | Intervention  N = 325 |
| 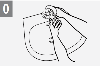Wet hands | 189 (100%) | 75 (100%) | 114 (100%) | 544 (84%) | 269 (84%) | 275 (85%) |
| 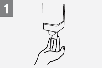Apply soap | 0 (0%) | 0 (0%) | 0 (0%) | 423 (66%) | 210 (65%) | 213 (66%) |
| 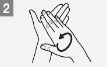Scrub: Palm to palm | 81 (43%) | 41 (55%) | 40 (35%) | 540 (84%) | 280 (88%) | 260 (80%) |
| 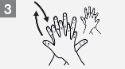Rub: Back of one hand against the palm of the other | 23 (12%) | 14 (19%) | 9 (8%) | 357 (55%) | 183 (57%) | 174 (54%) |
| 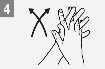Rub: Interfinger spaces | 46 (24%) | 19 (25%) | 27 (24%) | 227 (35%) | 114 (36%) | 113 (35%) |
| 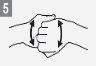Rubbing: Backs of fingers in the palm of the opposite hand | 0 (0%) | 0 (0%) | 0 (0%) | 82 (13%) | 51 (16%) | 31 (10%) |
| 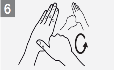Rubbing: Thumb of one hand rotated in the other hand | 0 (0%) | 0 (0%) | 0 (0%) | 52 (8%) | 27 (8%) | 25 (8%) |
| 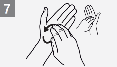Rubbing: Fingertips of one hand in the palm of the other hand | 3 (2%) | 1 (1%) | 2 (2%) | 141 (22%) | 80 (25%) | 61 (19%) |
| 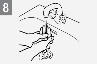Rinse hands with water | 127 (67%) | 61 (81%) | 66 (58%) | 373 (58%) | 196 (61%) | 177 (54%) |
| 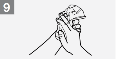Dry hands with a plain towel | 3 (2%) | 0 (0%) | 3 (3%) | 96 (15%) | 52 (16%) | 44 (14%) |
| 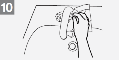Turn off the tap with the same towel | 41 (22%) | 3 (4%) | 38 (33%) | 45 (7%) | 22 (7%) | 23 (7%) |
