# Supplementary material for: Baseline Assessment of Handwashing Behavior, Hand Hygiene Conditions, and Wellbeing in Primary Schools in Nigeria
Source: Int J Public Health. 2025 Sep 25;70:1608656. doi: 10.3389/ijph.2025.1608656 (PMC12507709; doi:10.3389/ijph.2025.1608656)
Supplement: Supplementary file 1 [file DataSheet1.zip › Supplementary Table 8_revised.docx]

International Journal of Public Health

Baseline Assessment of Handwashing Behavior, Hand Hygiene Conditions, and Well-being in Primary Schools in Nigeria

## **Supplementary Table 8. Hand rinse sample results of children in intervention and control schools (Baseline assessment of handwashing behavior, hand hygiene conditions, and wellbeing in primary schools, Jere and Maiduguri Metropolitan Council, Nigeria, May–June 2023)**

| The hand rinse samples details | Overall  N = 311 | Control  N = 155 | Intervention  N = 156 |
| --- | --- | --- | --- |
| Duration from the time the sample was taken to the time of deposition on the plate | 311 | 155 | 156 |
|  |  |  |  |
| 3-6 hours | 294 (95%) | 142 (92%) | 152 (97%) |
| More than 6 hours | 17 (6%) | 13 (8%) | 4 (3%) |
| Countable EC counts | 178 | 90 | 88 |
| Mean (SD) | 94 (74) | 86 (72) | 103 (76) |
| Countable TC counts | 124 | 68 | 56 |
| Mean (SD) | 123 (81) | 128 (81) | 117 (82) |
| Uncountable EC | 133 | 65 | 68 |
| Not detectable (unclear for counting) | 26 (20%) | 21 (32%) | 5 (7%) |
| Too many to count | 107 (80%) | 44 (68%) | 63 (93%) |
| Uncountable TC | 187 | 87 | 100 |
| Not detectable (unclear for counting) | 75 (40%) | 36 (41%) | 39 (39%) |
| Too many to count | 112 (60%) | 51 (59%) | 61 (61%) |
| EC level (CFU/100mL) | 285 | 134 | 151 |
| None (0) | 3 (1%) | 3 (2%) | 0 (0%) |
| Low (1–10) | 13 (5%) | 7 (5%) | 6 (4%) |
| Moderate (11–100) | 95 (33%) | 54 (40%) | 41 (27%) |
| High (101–300) | 67 (24%) | 26 (19%) | 41 (27%) |
| Very high (> 300) | 107 (38%) | 44 (33%) | 63 (42%) |
| TC level (CFU/100mL) | 236 | 119 | 117 |
| None (0) | 7 (3%) | 3 (3%) | 4 (3%) |
| Low (1–10) | 5 (2%) | 2 (2%) | 3 (3%) |
| Moderate (11–100) | 44 (19%) | 24 (20%) | 20 (17%) |
| High (101–300) | 68 (29%) | 39 (33%) | 29 (25%) |
| Very high (> 300) | 112 (47%) | 51 (43%) | 61 (52%) |

*Abbreviations: EC: Escherichia coli, TC: Total coliform, CFU/100mL: colony-forming units/100 mL hand rinse samples*
