# Supplementary material for: Baseline Assessment of Handwashing Behavior, Hand Hygiene Conditions, and Wellbeing in Primary Schools in Nigeria
Source: Int J Public Health. 2025 Sep 25;70:1608656. doi: 10.3389/ijph.2025.1608656 (PMC12507709; doi:10.3389/ijph.2025.1608656)
Supplement: Supplementary file 1 [file DataSheet1.zip › Supplementary Table 9_revised.docx]

International Journal of Public Health

Baseline Assessment of Handwashing Behavior, Hand Hygiene Conditions, and Well-being in Primary Schools in Nigeria

## **Supplementary Table 9. Hygiene knowledge of children in intervention and control schools (Baseline assessment of handwashing behavior, hand hygiene conditions, and wellbeing in primary schools, Jere and Maiduguri Metropolitan Council, Nigeria, May–June 2023)**

| **N (%)** | | | |
| --- | --- | --- | --- |
| **Health knowledge** | Overall  N = 645 | Control  N = 320 | Intervention  N = 325 |
| *Children who believe that:* |  |  |  |
| Germs can stick to hands | 463 (72%) | 243 (76%) | 220 (68%) |
| Washing hands with water only is enough to remove germs | 407 (63%) | 197 (62%) | 210 (65%) |
| Children do not need to use soap | 183 (28%) | 87 (27%) | 96 (30%) |
| Cholera is only dangerous for children and not for adults | 250 (39%) | 125 (39%) | 125 (38%) |
| Touching dirty things transmits diseases | 601 (93%) | 295 (92%) | 306 (94%) |
| Germs can cause Cholera | 543 (84%) | 275 (86%) | 268 (82%) |
