# Supplementary material for: Baseline Assessment of Handwashing Behavior, Hand Hygiene Conditions, and Wellbeing in Primary Schools in Nigeria
Source: Int J Public Health. 2025 Sep 25;70:1608656. doi: 10.3389/ijph.2025.1608656 (PMC12507709; doi:10.3389/ijph.2025.1608656)
Supplement: Supplementary file 1 [file DataSheet1.zip › Supplementary Table 10_revised.docx]

International Journal of Public Health

Baseline Assessment of Handwashing Behavior, Hand Hygiene Conditions, and Well-being in Primary Schools in Nigeria

## **Supplementary Table 10. RANAS (Risks, Attitudes, Norms, Abilities and Self-regulation) hand hygiene behavioral factors of children in intervention and control schools (Baseline assessment of handwashing behavior, hand hygiene conditions, and wellbeing in primary schools, Jere and Maiduguri Metropolitan Council, Nigeria, May–June 2023)**

|  | **N (%)** | | |
| --- | --- | --- | --- |
|  | Overall  N = 645 | Control  N = 320 | Intervention  N = 325 |
| **Vulnerability** |  |  |  |
| *More than medium:* |  |  |  |
| Perceived risk of Cholera from not washing hands with soap before eating | 418 (65%) | 204 (64%) | 214 (66%) |
| Perceived risk of Cholera from not washing hands with soap after using the toilet | 374 (58%) | 184 (58%) | 190 (59%) |
| **Severity** |  |  |  |
| *More than medium:* |  |  |  |
| Perceived severity of Cholera on own life | 424 (66%) | 212 (66%) | 212 (65%) |
| Recalled severity of Cholera on any of the family member's life | 396 (61%) | 205 (64%) | 191 (59%) |
| **Attitude** |  |  |  |
| *More than medium:* |  |  |  |
| Perceived time consumption of handwashing | 149 (23%) | 73 (23%) | 76 (23%) |
| Perceived soap waste from handwashing | 209 (32%) | 106 (33%) | 103 (32%) |
| Perceived water waste from handwashing | 337 (52%) | 172 (54%) | 165 (51%) |
| Perceived cleanliness after handwashing with soap | 484 (75%) | 245 (77%) | 239 (74%) |
| Enjoying handwashing with soap before eating | 359 (56%) | 179 (56%) | 180 (55%) |
| Enjoying handwashing with soap after using the toilet | 214 (33%) | 100 (31%) | 114 (35%) |
| Perceived play interruption from handwashing before eating | 280 (43%) | 158 (49%) | 122 (38%) |
| Perceived study interruption from handwashing after toilet use | 247 (38%) | 141 (44%) | 106 (33%) |
| Preference for soap scent | 349 (54%) | 173 (54%) | 176 (54%) |
| Dislike of getting wet during handwashing | 141 (22%) | 74 (23%) | 67 (21%) |
| Enjoy playing when washing hands with soap | 418 (65%) | 225 (70%) | 193 (59%) |
| Dislike of cold water for handwashing | 179 (28%) | 81 (25%) | 98 (30%) |
| **Norms** |  |  |  |
| *More than half of the times:* |  |  |  |
| Perceived number of classmates washing hands with soap before eating | 32 (5%) | 13 (4%) | 19 (6%) |
| Perceived number of classmates washing hands with soap after toilet use | 27 (4%) | 5 (2%) | 22 (7%) |
| *More than medium:* |  |  |  |
| Perceived importance placed by teachers on handwashing with soap | 162 (25%) | 82 (26%) | 80 (25%) |
| Perceived peer pressure to handwashing with soap | 48 (7%) | 13 (4%) | 35 (11%) |
| Guilt felt for not washing hands after toilet use | 227 (35%) | 105 (33%) | 122 (38%) |
| Guilt felt for not washing hands before eating | 304 (47%) | 147 (46%) | 157 (48%) |
| **Abilities** |  |  |  |
| *More than medium:* |  |  |  |
| Confidence in consistently washing hands with soap | 285 (44%) | 128 (40%) | 157 (48%) |
| **Self-regulation** |  |  |  |
| Awareness of handwashing situations at school | 541 (84%) | 280 (88%) | 261 (80%) |
| Knowledge of handwashing facilities at school | 538 (83%) | 287 (90%) | 251 (77%) |
| Awareness of soap availability at school | 94 (15%) | 54 (17%) | 40 (12%) |
| *More than medium:*  Self-monitoring of handwashing with soap before eating | 272 (42%) | 127 (40%) | 145 (45%) |
| Self-monitoring of handwashing with soap after toilet use | 189 (29%) | 80 (25%) | 109 (34%) |
| *More than half of the times:*  Frequency of not washing hands due to unavailability of soap | 201 (31%) | 101 (32%) | 100 (31%) |
| Frequency of skipping handwashing to continue playing with friends | 206 (32%) | 111 (35%) | 95 (29%) |
| Frequency of avoiding handwashing due to dirty sink conditions | 124 (19%) | 60 (19%) | 64 (20%) |
| Frequency of not washing hands due to long queues | 151 (23%) | 79 (25%) | 72 (22%) |
| Frequency of forgetting handwashing before eating | 242 (38%) | 129 (40%) | 113 (35%) |
| Frequency of forgetting handwashing after toilet use | 343 (53%) | 179 (56%) | 164 (50%) |
