# Supplementary material for: Baseline Assessment of Handwashing Behavior, Hand Hygiene Conditions, and Wellbeing in Primary Schools in Nigeria
Source: Int J Public Health. 2025 Sep 25;70:1608656. doi: 10.3389/ijph.2025.1608656 (PMC12507709; doi:10.3389/ijph.2025.1608656)
Supplement: Supplementary file 1 [file DataSheet1.zip › Supplementary Table 11_revised.docx]

International Journal of Public Health

Baseline Assessment of Handwashing Behavior, Hand Hygiene Conditions, and Well-being in Primary Schools in Nigeria

## **Supplementary Table 11. Self-reported access to hand hygiene services at school** **of children in intervention and control schools (Baseline assessment of handwashing behavior, hand hygiene conditions, and wellbeing in primary schools, Jere and Maiduguri Metropolitan Council, Nigeria, May–June 2023)**

| **N (%)** | | | |
| --- | --- | --- | --- |
| **Self-reported access to hand hygiene services at school** | Overall  N = 645 | Control  N = 320 | Intervention  N = 325 |
| *More than half of the times frequency of the following characteristics:* |  |  |  |
| Sufficient handwashing water supply at school | 321 (50%) | 177 (55%) | 144 (44%) |
| Clean handwashing water at school | 376 (58%) | 195 (61%) | 181 (56%) |
| Acceptable color of handwashing water at school | 369 (57%) | 197 (62%) | 172 (53%) |
| Acceptable smell of handwashing water at school | 306 (47%) | 155 (48%) | 151 (46%) |
| Clear with no suspending particles handwashing water at school | 296 (46%) | 163 (51%) | 133 (41%) |
| *More than medium*  Satisfaction with school HWS | 243 (38%) | 134 (42%) | 109 (34%) |
