# Supplementary material for: Baseline Assessment of Handwashing Behavior, Hand Hygiene Conditions, and Wellbeing in Primary Schools in Nigeria
Source: Int J Public Health. 2025 Sep 25;70:1608656. doi: 10.3389/ijph.2025.1608656 (PMC12507709; doi:10.3389/ijph.2025.1608656)
Supplement: Supplementary file 1 [file DataSheet1.zip › Supplementary Table 12.docx]

International Journal of Public Health

Baseline Assessment of Handwashing Behavior, Hand Hygiene Conditions, and Well-being in Primary Schools in Nigeria

## **Supplementary Table 12. Self-reported quality of life (QoL) of children stratified by sex of children in schools (Baseline assessment of handwashing behavior, hand hygiene conditions, and wellbeing in primary schools, Jere and Maiduguri Metropolitan Council, Nigeria, May–June 2023)**

|  | **Mean (SD)** | | |
| --- | --- | --- | --- |
| **Quality of life** | Overall  N = 645 | Female  N = 388 | Male  N = 257 |
| Total Quality of Life | 65.2 (8.9) | 65.2 (9.2) | 65.1 (8.6) |
| Physical well-being | 70.0 (17.2) | 68.8 (17.8) | 71.8 (16.1) |
| Emotional well-being | 70.7 (17.1) | 70.2 (16.4) | 71.4 (18.1) |
| Self-esteem | 52.9 (24.0) | 54.3 (23.3) | 50.8 (24.8) |
| Family connection | 69.8 (17.4) | 69.7 (17.1) | 69.9 (18.0) |
| Friends (social well-being) | 70.2 (17.9) | 70.7 (18.1) | 69.6 (17.6) |
| Functioning at school | 57.4 (16.4) | 57.5 (16.6) | 57.3 (16.3) |
