# Supplementary material for: Baseline Assessment of Handwashing Behavior, Hand Hygiene Conditions, and Wellbeing in Primary Schools in Nigeria
Source: Int J Public Health. 2025 Sep 25;70:1608656. doi: 10.3389/ijph.2025.1608656 (PMC12507709; doi:10.3389/ijph.2025.1608656)
Supplement: Supplementary file 1 [file DataSheet1.zip › Supplementary Table 13.docx]

International Journal of Public Health

Baseline Assessment of Handwashing Behavior, Hand Hygiene Conditions, and Well-being in Primary Schools in Nigeria

**Supplementary Table 13. Self-reported quality of life (QoL) stratified by observed and self-reported handwashing behavior of children in schools (Baseline assessment of handwashing behavior, hand hygiene conditions, and wellbeing in primary schools, Jere and Maiduguri Metropolitan Council, Nigeria, May–June 2023)**

|  |  | **Mean (SD)** | | | | | | |
| --- | --- | --- | --- | --- | --- | --- | --- | --- |
| **Group** | N | Total Quality of life | Physical well-being | Emotional well-being | Self-esteem | Family connection | Friends (social well-being) | Functioning at school |
| Observed **washed** their hands **before eating** a snack during a structured opportunity (*primary outcome*) | 46 | 64.6 (9.1) | 71.7 (16.5) | 68.1 (15.5) | 51.6 (24.5) | 67.4 (17.1) | 68.2 (18.6) | 60.6 (15.6) |
| Observed **didn’t** wash their hands **before eating** a snack during a structured opportunity | 499 | 65.3 (8.7) | 69.7 (17.3) | 70.7 (17.1) | 54.1 (23.5) | 69.3 (17.5) | 70.5 (17.4) | 57.7 (16.5) |
|  |  |  |  |  |  |  |  |  |
| Observed washed their hands **after using the toilet** | 3 | 59.7 (8.9) | 62.5 (22.5) | 66.7 (9.5) | 47.9 (14.4) | 68.8 (12.5) | 70.8 (15.7) | 41.7 (20.1) |
| Observed **didn’t** wash their hands **after using the toilet** | 242 | 65.4 (8.8) | 69.3 (17.7) | 70.2 (17.2) | 55.1 (23.7) | 69.3 (17.0) | 71.4 (17.9) | 57 (15.8) |
|  |  |  |  |  |  |  |  |  |
| Self-reported handwashing frequency **before eating** (**more than half of the times**) | 172 | 65.8 (7.9) | 68.1 (17.5) | 72.3 (15.1) | 54.8 (22.6) | 70.3 (16.1) | 71.2 (17) | 58.2 (16.4) |
| Self-reported handwashing frequency **before eating** (**half or less than half of the times**) | 473 | 64.9 (9.2) | 70.7 (17.0) | 70.1 (17.7) | 52.2 (24.4) | 69.6 (17.9) | 69.9 (18.2) | 57.1 (16.5) |
|  |  |  |  |  |  |  |  |  |
| Self-reported handwashing frequency **after toilet use** (**more than half of the times**) | 78 | 66.2 (8.2) | 69.2 (19.2) | 72.9 (15.9) | 51.8 (22.5) | 72 (17.9) | 71.7 (18.1) | 59.5 (15.9) |
| Self-reported handwashing frequency **after toilet use** (**half or less than half of the times**) | 567 | 65.0 (9.0) | 70.1 (16.9) | 70.4 (17.2) | 53.1 (24.2) | 69.5 (17.4) | 70 (17.9) | 57.1 (16.5) |
